# Motion Tape Strain During Trunk Muscle Engagement in Young, Healthy Participants

**DOI:** 10.3390/s24216933

**Published:** 2024-10-29

**Authors:** Spencer Spiegel, Elijah Wyckoff, Jay Barolo, Audrey Lee, Emilia Farcas, Job Godino, Kevin Patrick, Kenneth J. Loh, Sara P. Gombatto

**Affiliations:** 1 Department of Mathematics and Statistics, San Diego State University, San Diego, CA 92182, USA; 2 Department of Structural Engineering, University of California San Diego, La Jolla, CA 92093, USA; ewyckoff@ucsd.edu (E.W.);; 3 School of Exercise and Nutritional Sciences, San Diego State University, San Diego, CA 92182, USA; 4 Department of Bioengineering, San Diego State University, San Diego, CA 92182, USA; alee5285@sdsu.edu; 5 Qualcomm Institute, University of California San Diego, La Jolla, CA 92093, USAkpatrick@ucsd.edu (K.P.); 6 Laura Rodriguez Research Institute, Family Health Centers of San Diego, San Diego CA 92102, USA; 7 School of Public Health, University of California San Diego, La Jolla, CA 92093, USA; 8 School of Physical Therapy, San Diego State University, San Diego, CA 92182, USA

**Keywords:** sensor, wearable, body-worn sensors, nanocomposite, textile, low back, muscle activity

## Abstract

Background: Motion Tape (MT) is a low-profile, disposable, self-adhesive wearable sensor that measures skin strain. Preliminary studies have validated MT for measuring lower back movement. However, further analysis is needed to determine if MT can be used to measure lower back muscle engagement. The purpose of this study was to measure differences in MT strain between conditions in which the lower back muscles were relaxed versus maximally activated. Methods: Ten participants without low back pain were tested. A matrix of six MTs was placed on the lower back, and strain data were captured under a series of conditions. The first condition was a baseline trial, in which participants lay prone and the muscles of the lower back were relaxed. The subsequent trials were maximum voluntary isometric contractions (MVICs), in which participants did not move, but resisted the examiner force in extension or rotational directions to maximally engage their lower back muscles. The mean MT strain was calculated for each condition. A repeated measures ANOVA was conducted to analyze the effects of conditions (baseline, extension, right rotation, and left rotation) and MT position (1–6) on the MT strain. Post hoc analyses were conducted for significant effects from the overall analysis. Results: The results of the ANOVA revealed a significant main effect of condition (*p* < 0.001) and a significant interaction effect of sensor and condition (*p* = 0.01). There were significant differences in MT strain between the baseline condition and the extension and rotation MVIC conditions, respectively, for sensors 4, 5, and 6 (*p* = 0.01–0.04). The largest differences in MT strain were observed between baseline and rotation conditions for sensors 4, 5, and 6. Conclusions: MT can capture maximal lower back muscle engagement while the trunk remains in a stationary position. Lower sensors are better able to capture muscle engagement than upper sensors. Furthermore, MT captured muscle engagement during rotation conditions better than during extension.

## 1. Introduction

Low back pain (LBP) is one of the most burdensome health conditions worldwide and is only increasing in prevalence [1]. Between 1990 and 2017 there was a 53% increase in years lived with disability due to LBP [2]. In a systematic analysis of the global burden of LBP, 619 million cases of LBP were reported globally in 2020, and projections indicated that the number of cases will increase to 843 million by 2050 [3]. In addition to impacting quality of life, LBP poses a significant financial burden. In the United States, between 12 and 91 billion U.S. dollars are spent annually on the management of LBP [4]. There is also a massive loss of productivity associated with LBP, with the highest indirect cost associated with LBP resulting from lost work productivity [4]. As the global prevalence of LBP increases [1], there is a need for improved assessments and interventions to address this burdensome health condition.

Altered activation of trunk muscles is a common impairment in people with LBP compared to healthy controls, including reduced muscle activation and force generation during activities [5], greater muscle activation during static standing [6], or altered motor control that can result in inefficient muscular stabilization of the spine [7,8]. These alterations in the patterns of lower back muscle activation in people with LBP suggest that tools for measuring muscle engagement would be valuable for the diagnosis and treatment of LBP. Specifically, a cost-effective method for identifying imbalances in muscle engagement across daily activities such as standing, sitting, walking, lifting, and bending could be useful for the diagnosis and management of LBP [6,9].

Currently, the most common method used to measure muscle engagement is electromyography (EMG). Although EMG is a useful tool, it has multiple drawbacks. EMG sensors are expensive, require expertise to use and interpret, and are typically limited to a laboratory setting. These limitations prevent practicing clinicians from using EMG in a clinical setting or monitoring muscle engagement in patients with LBP in a free-living environment. Because people behave differently when they know they are being observed (i.e., the “Hawthorne Effect”), measuring muscle engagement outside of the laboratory may be critically important to capture alterations that are associated with LBP [10]. However, EMG is not currently well suited for use in this environment.

In reviews of wearable technology used for measuring aspects of LBP, most existing sensors capture movement rather than muscle engagement, and few can be used in a free-living environment [11,12]. Additionally, many existing sensors are rigid and do not fit well with the curvatures and multi-segmental nature of the lower back. To address these limitations, various on-skin sensor technologies are being developed and tested for human–machine interfaces [13], as well as healthcare applications [14]. Motion Tape (MT) is an emerging on-skin sensor technology that has been validated for measuring joint movement and muscle engagement in the upper extremity and ankle [15,16,17]. Motion Tape is a low-cost, unobtrusive device that has the potential to capture muscle engagement in a clinical or free-living environment. Recently, a lower back use case was developed. A preliminary laboratory validation study was conducted and demonstrated that MT can accurately measure movement in the low back [18]. The usability and acceptability of MT for the low back use case was examined for both “patient” and Physical Therapist users, and MT was deemed acceptable by both user groups, although suggestions for future improvement of sensors were identified [18,19]. However, the extent to which MT can measure lower back muscle engagement has not previously been evaluated.

The purpose of this study was to investigate the extent to which MT can measure lower back muscle engagement, specifically during maximum voluntary isometric contraction (MVIC) tests, which involve engagement of lower back muscles but little to no lower back movement. We hypothesized that MT would measure greater strain during MVIC tests compared to a prone position when the lower back muscles are relaxed.

## 2. Methods

### 2.1. Participants

Participants were recruited from a college campus using email flyers sent to students, faculty, and staff. A sample size of 10 participants was considered adequate for a preliminary validation study of MT to measure muscle engagement and provide a basis for improvement of the prototype device.

People between the ages of 18–65 years with no reported history of LBP within the last year were eligible for participation. Participants were excluded if they were (1) unable to follow instructions given in English, (2) unable to perform the required movements, or (3) unwilling to wear tight-fitting shorts and a sports bra (women) or no shirt (men). Recruitment and testing occurred during the period from January 2023 to March 2023. Data were collected in the Rehabilitation Biomechanics Laboratory at San Diego State University (SDSU). The study was approved by the SDSU Institutional Review Board (IRB#HS-2022-0269), and each participant provided their written informed consent before participating.

### 2.2. Motion Tape

Motion Tape (MT) derives its strain-sensing properties from the piezoresistive properties of the integrated and percolated graphene nanosheet (GNS) nanocomposite morphology within the elastic fabric substrate of commercial kinesiology tape (Rock Tape, Durham, NC, USA) [15,20]. In order to manufacture MT, a solution is produced by combining GNSs and ethyl cellulose (EC) in an ethyl alcohol solution [15,20]. All solvents are purchased from Sigma-Aldrich (St. Louis, MO, USA). Dispersion of the GNSs in 2 wt% EC is achieved using bath and high-energy probe sonication. Then, the dispersed GNS-EC solution is spray-coated onto a masked region of the kinesiology tape to form a 4 × 1 cm^2^ nanocomposite. Spray-coating is repeated three times to achieve a target baseline (or nominal) resistance of the GNS-EC nanocomposite to be approximately 10 kΩ. Should the baseline resistance fail to reach this value, an additional layer is drop-casted to decrease its electrical resistance. 

After the application and drying of the layers, colloidal silver paste is applied at each end of the nanocomposite to create conductive terminals. Conductive wires are soldered onto these silver paste terminals to facilitate strain measurements. These wires are connected to a custom-printed circuit board (PCB) with a Bluetooth transmitter. The collected signals are transmitted via Bluetooth to the MT Data Acquisition (DAQ) 2.2 board (equipped with a CC1350 microcontroller, Texas Instruments, Dallas, TX, USA). The MT DAQ connects to a laptop computer via a micro-USB cable, where test data are recorded and labeled in SmartRF Studio 7.3 (Texas Instruments). More details about the design of the MT DAQ are reported in Pierce et al. [21]. In addition, previous research has demonstrated that MT exhibits stable performance under cyclic strains of low magnitude, including high linearity, repeatability, and minimal hysteresis, even after more than 100 cycles of loading [20].

MT data are captured as electrical resistance measured in ohms (Ω), with labeled timestamps and a sampling rate of approximately 65 Hz. The normalized resistance change (*R_n_*) is calculated using Equation (1):

(1) $$R_{n}=\frac{R-R_{0}}{R_{0}}$$

where *R* is the electrical resistance of MT at any given time instance, and *R*_0_ is its nominal or baseline resistance. Nominal resistance is defined as the initial electrical resistance of MT after it has been affixed onto the skin and when the participant is in a relaxed, natural posture. In this work, *R_n_* was processed using a method of locally weighted scatterplot smoothing, or Lowess, to reduce noise. The Lowess method defines a regression weight function for the data points contained within the span, which is defined as 10% of the span for the MT resistance data.

### 2.3. Motion Tape Placement

**Figure 1:**
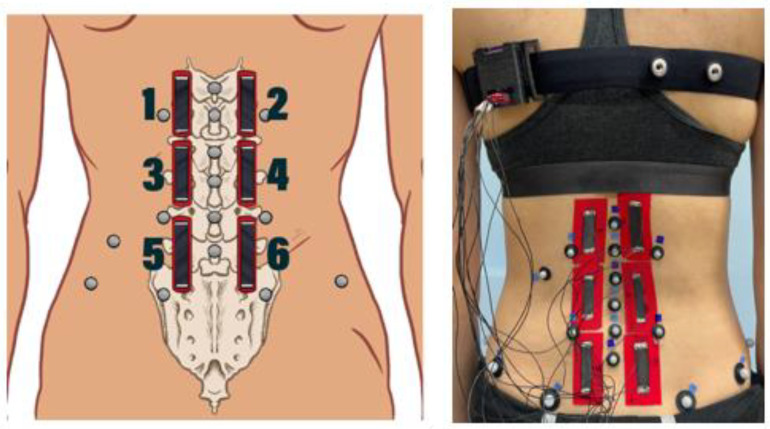
Motion Tapes (1–6) and motion capture marker placement schematic (**left**) and on an actual participant (**right**) [9].

MT was used to measure muscle engagement during test conditions. Six MTs were placed on the low back, lateral to the spinal column and patterned in a 3 × 2 matrix (Figure 1). This configuration was selected to monitor three different regions of the low back (upper, lower, lumbopelvic) and to parallel the EMG placement and motion capture sensor placement from an existing spine model [22,23,24]. The use of three pieces of tape on each side of the low back also allows for more freedom of movement relative to a single piece of tape on each side of the low back. This configuration was validated for measuring low back movement in a previous study [18]. The same Physical Therapist clinician-researcher (SPG), with 20+ years of experience in optical motion capture of the spine, performed all placements. The placement of the MTs started with the middle MTs (sensors 3 and 4) to ensure that the bottom edges of the middle MTs were placed at a level just above the L4 spinous process and crossed the L2-L3 and L3-L4 junctions for most (90%) of the participants. The superior MTs (sensors 1 and 2) were placed above the middle MTs to ensure that the superior MTs crossed the T12-L1 and L1-L2 junctions for most (90%) of the participants. The inferior MTs (sensors 5 and 6) were placed below the middle tapes so that the inferior MTs crossed the L4-L5 and L5-S1 junctions for most (90%) of the participants.

### 2.4. Three-Dimensional Optical Motion Capture

Optical motion capture was used to measure low back movement during test conditions as a methodological check to confirm that there was minimal movement. Retroreflective optical motion capture markers were placed on the T12-L5 spinous processes and bilaterally to the left and right of L1 and L4 at approximately 4 cm from the spinal column (see Figure 1). The markers were used to create a modified version of a previously validated multi-segmental spine model for measuring lumbar spine posture and movement [23]. The upper lumbar segment was defined by a single marker on the spinous process of L3 and the lateral markers to the left and right of L1. The lower lumbar segment was defined by a single marker on the spinous process of L5 and markers to the left and right of L4. The pelvis segment was defined by markers placed bilaterally on the posterior superior iliac spine, anterior superior iliac spine, posterior pelvis, and iliac crests.

### 2.5. MVIC Testing Protocol

In prior studies in other body regions, MT measures of muscle engagement were compared to simultaneous EMG measures of muscle activity [15]. For use on the low back, MT is placed over muscles of the low back in the same location that EMG sensors would be placed [18]. Therefore, we could not use EMG sensors as a reference standard for testing low back muscle engagement simultaneously with MT. As an alternative, the current study protocol was designed to evaluate MT strain during a condition in which low back muscles were engaged, but movement was minimized. A maximum voluntary isometric contraction (MVIC) test is a commonly used test for maximal low back muscle engagement, with little to no low back movement [25].

MT strain data were captured under four different conditions. All conditions were designed so that the participants had little to no movement of the lumbar spine region. The first condition was baseline (BASE), in which a 1 s trial captured the participant lying prone on a treatment table with the muscles of the low back fully relaxed. The remaining 3 conditions were MVICs in which the participant engaged the muscles of the low back against maximal examiner resistance without moving for a 5 s trial. The trials included MVIC extension (EXT) and MVIC left/right rotation (LROT/RROT). All conditions are described in greater detail and depicted visually in Table 1. The following approaches were used to ensure consistency of implementation of the study protocol: (1) a consistent experimental setup (table, belts for stabilization, and participant and examiner position) was used; (2) a script was used for participant instructions; (3) the same examiner, a Physical Therapist with 20+ years of experience in LBP research, conducted all testing; and (4) methodologic confirmation that MVIC tests were isometric (e.g., little to no movement) was achieved using 3D optical motion capture.

### 2.6. Data Processing

**Figure 2:**
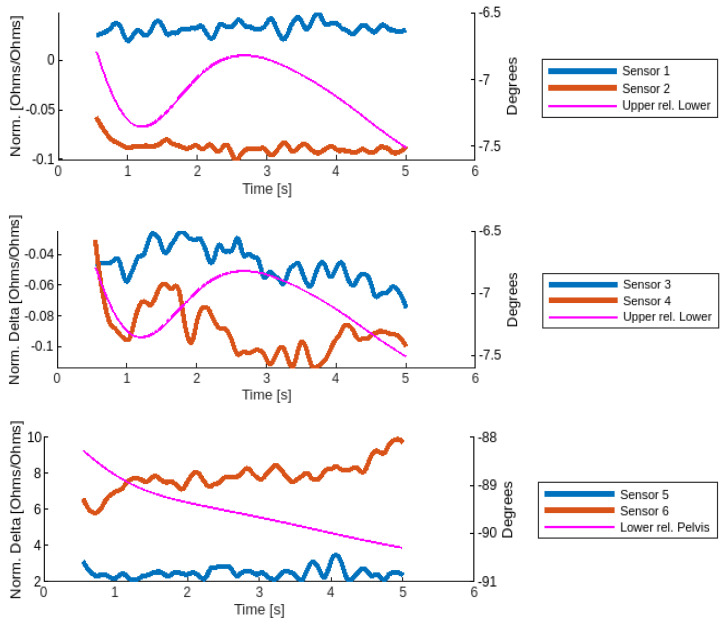
Motion Tape strain data (sensors 1–6) and lumbar kinematic data for a representative participant during a left rotation (LROT) MVIC trial.

The Motion Tape strain and kinematic data for a representative participant during an MVIC LROT trial is displayed in Figure 2. The raw data from the MT were processed as described previously [18]. Briefly, the time series data for MT strain were downsampled to ensure that all trials were time series’ of identical lengths (Figure 2). The mean MT strain was then calculated across the time series, providing an average strain value for each condition and each participant.

Low back kinematics were derived from motion capture markers to confirm that the MVIC trials involved minimal low back movement. These data were processed as described previously by applying a multi-segmental spine model and calculating the kinematic angles between the upper lumbar, lower lumbar, and pelvis segments [18]. The angle between the upper lumbar segment (L1-L3) and the lower lumbar segment (L4-L5), as well as between the lower lumbar segment (L4-L5) and the pelvis segment, were calculated for each participant across the duration of each trial (Figure 2). The excursion of each lumbar segment was calculated across the time series by subtracting the minimum angle from the maximum angle for each condition and for each participant. Thus, the excursion reflects the extent to which the lumbar segment moved during the trial. This excursion measure was used as a methodological check to confirm that there was minimal low back movement during the MVIC trial to ensure that the MT strain during each condition was primarily the result of muscle engagement and not due to low back movement.

### 2.7. Data Analysis

The average and standard deviation of lumbar segment excursions were calculated for each condition across all participants. The average and standard deviation for the mean MT strain were then calculated for each sensor, during each condition, across all participants.

To test whether MTs can measure low back muscle engagement, a within-subjects repeated measures ANOVA was performed to examine differences in mean MT strain across conditions of muscle engagement (BASE, EXT, LROT, RROT) for different MT sensors (1–6) and for the interaction between condition and sensor. Post hoc tests were conducted for significant effects to examine which sensors and conditions displayed significant differences in strain. Significant differences in MT strain between the BASE condition and the EXT, LROT, and RROT conditions would provide evidence that MT can measure muscle engagement during these conditions. An alpha level of 0.05 was used for all statistical tests.

## 3. Results

Ten people participated in this study, including five males (23.6 ± 2.9 years old) and five females (21.2 ± 1.8 years old). The mean and standard deviation values for lumbar segment excursions are reported in Table 2, confirming minimal movement during the MVIC conditions.

**Figure 3:**
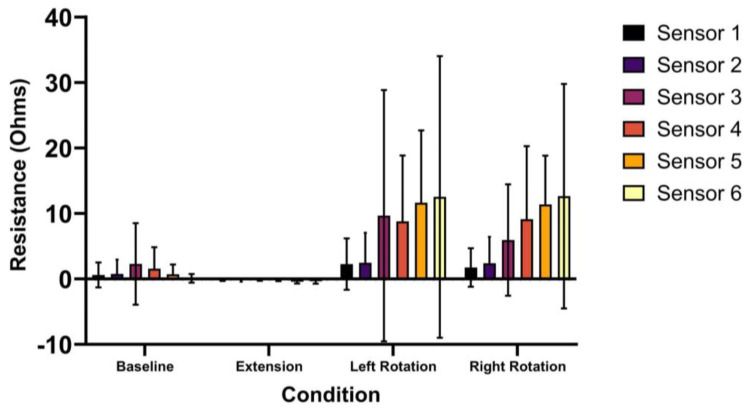
Average (standard deviation) strain (resistance in Ω) across all participants for Motion Tapes (sensors) 1–6 during each condition.

The values of the MT strain (resistance in Ω) for each sensor and condition are presented in Table 3. The results of the ANOVA indicated that there was a significant main effect of condition (*p* < 0.001) and a significant interaction effect of sensor and condition (*p* = 0.01) (Figure 3). Higher mean strain values were observed during MVIC left and right rotation compared to the baseline, while lower mean strain values were observed during the MVIC extension condition (Figure 3).

When exploring the significant interaction effect of sensor and condition found in the ANOVA, the post hoc testing revealed that differences in condition depended on which sensor was being evaluated. Sensors 1–3 did not display a significant effect of condition (*p* = 0.06–0.09), while a significant condition effect was present for sensors 4–6 (*p* ≤ 0.001–0.014). For sensor 4, there was a significantly greater strain during both MVIC LROT (*p* = 0.021) and MVIC RROT (*p* = 0.024) conditions compared to the baseline. For sensor 5, the strain from MVIC EXT was significantly lower than the baseline (*p* = 0.039), while those from MVIC LROT and MVIC RROT were significantly greater than the baseline (*p* = 0.001–0.011). For sensor 6, the strain from MVIC EXT (*p* = 0.018) was significantly lower than the baseline, and the strain from MVIC RROT (*p* = 0.046) was significantly higher than the baseline.

## 4. Discussion

The extent to which MT can measure low back muscle engagement during MVIC conditions depends on the condition and sensor placement. Sensors 1–3 did not measure a significant change in strain with MVIC conditions compared to the baseline, while sensors 4-6 displayed significant differences in strain during MVIC conditions compared to the baseline. Specifically, sensors 4–6 measured notably more strain during MVIC rotation conditions compared to the baseline, while sensors 5 and 6 measured less strain during the MVIC extension condition compared to the baseline. It is possible that an increase in strain during rotation versus a decrease in strain during extension MVICs could result from differences in skin surface change during lower lumbar muscular effort that varies based on the activity. For example, the positive strain with rotation conditions may result from the stretching of the skin during muscle contractions as the body resists torsion, while the negative strain may be associated with approximation of the skin as the muscles contract during the extension condition.

### 4.1. Comparison to Existing Technology

In prior investigations, MT has demonstrated the ability to identify unique movements [26] and muscle engagement in other body regions [15,16,17]. A previous study showed that repeating the same movement with increasing weights resulted in an increase in the strain measurements recorded by MT placed on the bicep muscle [15]. These findings suggest that MT strain values may be associated with the added skin stretch corresponding with greater muscle activation. The current study demonstrates that the skin strain of low back muscles depends on the direction of resistance. Future studies could examine whether the change in strain with low back muscle engagement varies based on the magnitude of the load applied.

In a previous study, our research group demonstrated a moderate to high association between MT strain and low back movement during dynamic trunk movements [18]. When examining the change in strain in the current study with MVIC conditions, which involve little to no movement, compared to the change in strain during dynamic trunk movements from the prior study, the differences varied based on the sensor and condition. For rotation conditions, sensors 1–4 displayed an average of 0.3–2.3 Ω higher strain during MVIC conditions compared to dynamic movements, whereas sensors 5 and 6 displayed 1.3–3.5 Ω lower strain during MVIC conditions compared to dynamic movements. For extension conditions, the change in strain was uniformly smaller (range of 0.2–1.8 Ω) during the MVIC condition than with dynamic extension movements. These results indicate that the skin stretching measured by MT is likely a combination of movement and muscle engagement. Future research to investigate the relative contributions of dynamic movement versus muscle engagement to MT low back strain could be conducted using indwelling electrodes for monitoring muscle engagement or could be estimated using musculoskeletal modeling. Understanding the relative contributions of muscle engagement and movement to MT low back strain may be important for identifying and addressing these different mechanisms of LBP.

A previous study using EMG to measure muscle engagement during MVIC conditions recorded more activation of low back muscles during lumbar extension than all other recorded movements [27]. These findings suggest that MVIC extension results in a significant amount of muscular engagement. However, in the current study, the MT recorded minimal strain during the MVIC extension condition. One possible explanation is that MT measures skin surface stretching resulting from muscle engagement, but for some movements such as MVIC extension, there is minimal skin stretching (or rather skin approximation). Thus, the strain captured by MT may not correspond to the underlying muscle engagement for lumbar extensor muscles.

### 4.2. Clinical Implications

This study indicates that MT can provide insights into low back muscle engagement, particularly for muscles that are involved with trunk rotation. Information about muscle engagement could be useful for healthcare providers when diagnosing and managing altered motor control in people with LBP [6,9,28]. In addition, because of its low cost, low profile, and portability, the potential for MT to capture muscle engagement in a free-living environment is particularly promising. While other sensors have been used to provide feedback to patients on muscle activation in a free-living environment as part of a clinical trial intervention, the sensors used were not validated for measuring muscle engagement [29].

Because of the low profile of MT and its ability to measure both rotation movement [18] and muscle engagement, it may be useful for monitoring the low back muscles during rotational activities associated with sports such as throwing, hitting a volleyball, or swinging in tennis, golf, or baseball. MT has previously been shown to be capable of quantifying complex rotational and extension movements during marksmanship exercises [30] and golf [26].

### 4.3. Limitations

A limitation of this study is the exclusive use of MVIC conditions to measure muscle engagement. While everyday activities often involve both muscle activation and movement of the lower back, it is methodologically challenging to decouple the strain that results from movement versus that of muscle activation. Simultaneous monitoring of muscle engagement with EMG and MT was not possible, because both sensors need to be positioned in the same location of the low back. Therefore, the current study proposed to measure the contribution of muscle engagement alone by limiting low back movement with the MVIC conditions. However, it may still be useful to be able to quantify the collective strain applied to the low back during everyday activities, irrespective of whether it is the result of muscle engagement or movement.

A second limitation of the study was that the sample size was small and included young, relatively fit, and injury-free participants with relatively homogenous body types. Because age changes skin characteristics, skin stretching associated with muscle engagement may be different for older people, which could impact the MT strain measurements. Similarly, higher amounts of subcutaneous fat in the lower back region could alter the skin stretch associated with muscle engagement. Last, some people with LBP may activate muscles less than people without LBP [5], and therefore, it may be more difficult to capture the strain resulting from muscle engagement with MT in some people with LBP. Future studies would need to include a larger sample size with people of different body types and ages, as well as people with LBP, to investigate the extent to which age, body composition, and the presence of LBP influence MT strain measures during low back muscle engagement. A larger sample would also be required to explore gender differences in MT strain measures.

Last, because the device is in a prototype state, there are wires that connect the MTs to the wireless data acquisition device. Future sensor developments should explore a wire-free MT design, which could better facilitate the use of MT in a free-living environment.

## Figures and Tables

**Table 1:** Description and illustration of the baseline and maximum voluntary isometric contraction (MVIC) conditions.

Condition	Description	Visual
Baseline (BASE)	Participant is prone on the treatment table with low back muscles relaxed.	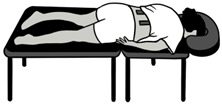
MVIC Extension (EXT)	Participant is prone with the pelvis supported on the treatment table up to the level of the anterior superior iliac spine. The participant holds the trunk level with the table in a neutral position against maximal examiner resistance on the upper back.	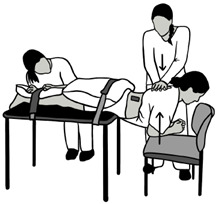
MVIC Left/Right Rotation (LROT/RROT)	Participant is seated upright at the edge of the treatment table, with feet supported on the ground and arms crossed. Participant pushes against maximal examiner resistance into the left rotation direction. This was repeated for the right rotation direction.	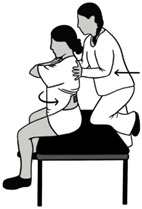

**Table 2:** Average (standard deviation) lumbar segment excursion (in degrees) during baseline and maximum voluntary isometric contraction (MVIC) conditions.

	Baseline (BASE)	MVIC Extension (EXT)	MVIC Left Rotation (LROT)	MVIC Right Rotation (RROT)
Upper relative to Lower (degrees)	1.0° (1.2°)	3.3° (2.8°)	3.3° (4.7°)	2.3° (2.1°)
Lower relative to Pelvis (degrees)	1.1° (1.9°)	3.3° (1.9°)	2.1° (3.0°)	1.7° (1.1°)

**Table 3:** Mean and standard deviation strain (resistance in Ω) for each sensor and condition across all participants (* significant effect of condition compared to baseline for each sensor from post hoc testing (*p* < 0.05)).

	BASE	EXT	LROT	RROT
Sensor 1	0.6 (1.9)	−0.1 (0.1)	2.3 (3.9)	1.8 (2.9)
Sensor 2	0.8 (2.2)	−0.2 (0.2)	2.5 (4.6)	2.4 (4.0)
Sensor 3	2.3 (6.2)	−0.1 (0.1)	9.7 (19.2)	6.0 (8.5)
Sensor 4	1.6 (3.3)	−0.1 (0.3)	8.8 * (10.1)	9.1 * (11.2)
Sensor 5	0.7 (1.5)	−0.4 * (0.3)	11.6 * (11.0)	11.4 * (7.5)
Sensor 6	0.1 (0.7)	−0.4 * (0.4)	12.5 (21.5)	12.6 * (17.1)

## Data Availability

The data presented in this study are available on request from the corresponding author.
